# Spatiotemporal change and prediction of land use in Manasi region based on deep learning

**DOI:** 10.1007/s11356-023-27826-0

**Published:** 2023-06-19

**Authors:** Jiaojiao Wang, Xiaojun Yin, Shannan Liu, Dimeng Wang

**Affiliations:** 1 College of Information Science & Technology, Shihezi University, Shihezi, China; 2 Geospatial Information Engineering Research Center, Xinjiang Production and Construction Corps, Shihezi, China

**Keywords:** Spatiotemporal change, Land use prediction, Deep learning, Landscape indices, MLP-LSTM

## Abstract

The Manasi region is located in an arid and semi-arid region with fragile ecology and scarce resources. The land use change prediction is important for the management and optimization of land resources. We utilized Sankey diagram, dynamic degree of land use, and landscape indices to explore the temporal and spatial variation of land use and integrated the LSTM and MLP algorithms to predict land use prediction. The MLP-LSTM prediction model retains the spatiotemporal information of land use data to the greatest extent and extracts the spatiotemporal variation characteristics of each grid through a training set. Results showed that (1) from 1990 to 2020, cropland, tree cover, water bodies, and urban areas in the Manasi region increased by 855.3465 km^2^, 271.7136 km^2^, 40.0104 km^2^, and 109.2483 km^2^, respectively, whereas grassland and bare land decreased by 677.7243 km^2^ and 598.5945 km^2^, respectively; (2) Kappa coefficients reflect the accuracy of the mode’s predictions in terms of quantity. The Kappa coefficients of the land use data predicted by the MLP-LSTM, MLP-ANN, LR, and CA-Markov models were calculated to be 95.58%, 93.36%, 89.48%, and 85.35%, respectively. It can be found that the MLP-LSTM and MLP-ANN models obtain higher accuracy in most levels, while the CA–Markov model has the lowest accuracy. (3) The landscape indices can reflect the spatial configuration characteristics of landscape (land use types), and evaluating the prediction results of land use models using landscape indices can reflect the prediction accuracy of the models in terms of spatial features. The results indicate that the model predicted by MLP-LSTM model conforms to the development trend of land use from 1990 to 2020 in terms of spatial features. This gives a basis for the study of the Manasi region to formulate relevant land use development and rationally allocate land resources.

## Introduction

Land overexploitation leads to ecosystem degradation, environmental pollution (Alqadhi et al. 2021), greening reduction, soil erosion, and biodiversity loss (Shafizadeh-Moghadam et al. 2019; Lukas et al. 2023), which affect economic development (Permatasari et al. 2021). Land use change is influenced by both human activities and natural environmental changes (Hasan et al. 2020). Therefore, land use prediction is of great significance to ecological protection and economic development. With the increasing integration of natural resources and the increasingly complex structure of land use, land use prediction is becoming highly complicated. The analysis of the evolution characteristics and land use prediction is not only conducive to understanding the relationship between land use and natural as well as social factors, but also provides a basis for effective land use planning (Yan et al. 2020). Furthermore, predicting future land use will help relevant personnel formulate reasonable ecological conservation and land use management strategies and promote the sound development of both ecology and the economy, which has important implications for managing resources.

Recently, deep learning techniques like random forest regression (Giang et al. 2022), convolutional neural network (CNN) (Zhang et al. 2021a), and Markov (Michel et al. 2021) cellular automata-Markov (CA-Markov) were applied to predict spatial distribution of land use (Chen et al. 2022). Deep learning models are prevalent in land use change predictions. Deep learning can fully utilize historical data for iterative training, which is suitable for complex land use prediction problems, such as the long-short-term memory model to predict land use (Mu et al. 2019). Some scholars have used various land use prediction models, including the cellular automata (CA) (Xing et al. 2020; Da Cunha et al. 2021), the FLUS (Liu et al. 2022), and the PLUS (Li et al. 2022; Lin and Peng 2022) models. Notably, the artificial neural network has been utilized for the analysis and predict future land use situations (Ansari and Golabi 2019; Pandey and Kumari 2023). The land change model (LCM) in the Idrisi GIS software (based on ANN and Markov chain) was used to analyze and predict future land use situations (Ansari and Golabi 2019; Pandey and Kumari 2023). The deep learning cellular automata (DL-CA) model was used to predict land use (Xing et al. 2020). Different land use models, including multilayer perceptron Markov chain, logistic regression-Markov model, multi-layer perceptron Markov chain cellular automata, and logistic regression Markov model cellular automata models were compared (Sankarrao et al. 2021).

The perceptron is a model of a single neuron and is the pioneer to larger neural network. In multi-class problems, multi-layer perceptron long-short-term memory (MLP-LSTM) uses more perceptrons, one perceptron per land-use type. The output of each perceptron indicates the probability of belonging to a category, and the category with the highest probability is the final output. MLP-LSTM model uses memory cells and gate mechanisms to control the transmission of sequence information and fully extracts the correlation information of time series data, enabling it to solve nonlinear and complex problems (Zhang et al. 2021b). By designing forget, input, and output gate, MLP-LSTM reduces or adds information to the cell unit. The forget gate filters out unimportant information that can be stored for an extended period. The input gate determines the content of the current data input that needs to be stored in the cell unit. The MLP-LSTM model retains the spatiotemporal information of land use data to the fullest extent and extracts the spatiotemporal variation characteristics of each grid through a training set.

CA-Markov, MLP-ANN, and LR have been used by many scholars to predict land use. Therefore, these three land use prediction models are selected to compare with the algorithm proposed in this paper in terms of accuracy and landscape index. The present study compared and analyzed four land use change prediction models. These models include the MLP-LSTM, multi-layer perception artificial neural network (MLP-ANN), and the LR and CA-Markov models. The objectives of this study can be categorized as follows: (1) The temporal and spatial patterns and the evolution characteristics of land use were analyzed using the Sankey diagram, dynamic degree of land use, and landscape indices. (2) The sensitivity of land use drivers was analyzed by combining the OLS and Sobol methods. (3) The LSTM and MLP algorithms were integrated to predict land use.

**Fig. 1:**
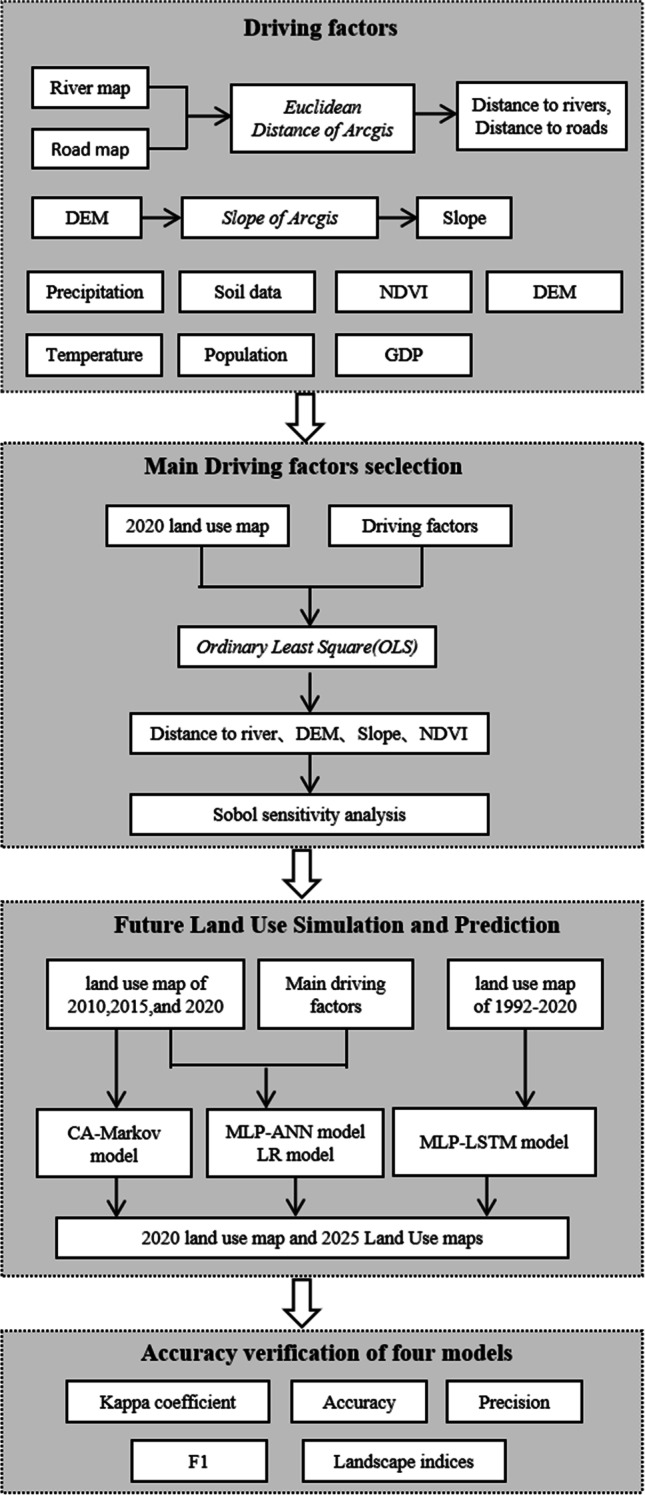
Schematic diagram for simulating the future land use by the MLP-LSTM, MLP-ANN, LR, and CA–Markov model

The study was conducted in four main phases (Fig. 1). In the first phase, land use data and driving factors were selected. Then, the ordinary least square (OLS) method in ArcGIS Pro was used to assess the drivers of land use change in the study area. In the third phase, four land use prediction models were employed to predict land use change in 2025. Finally, various accuracy assessment methods including Kappa, Accuracy, Precision, F1, and landscape indices were used to verify the accuracy of the models.

## Materials and methods

### Study area

**Fig. 2:**
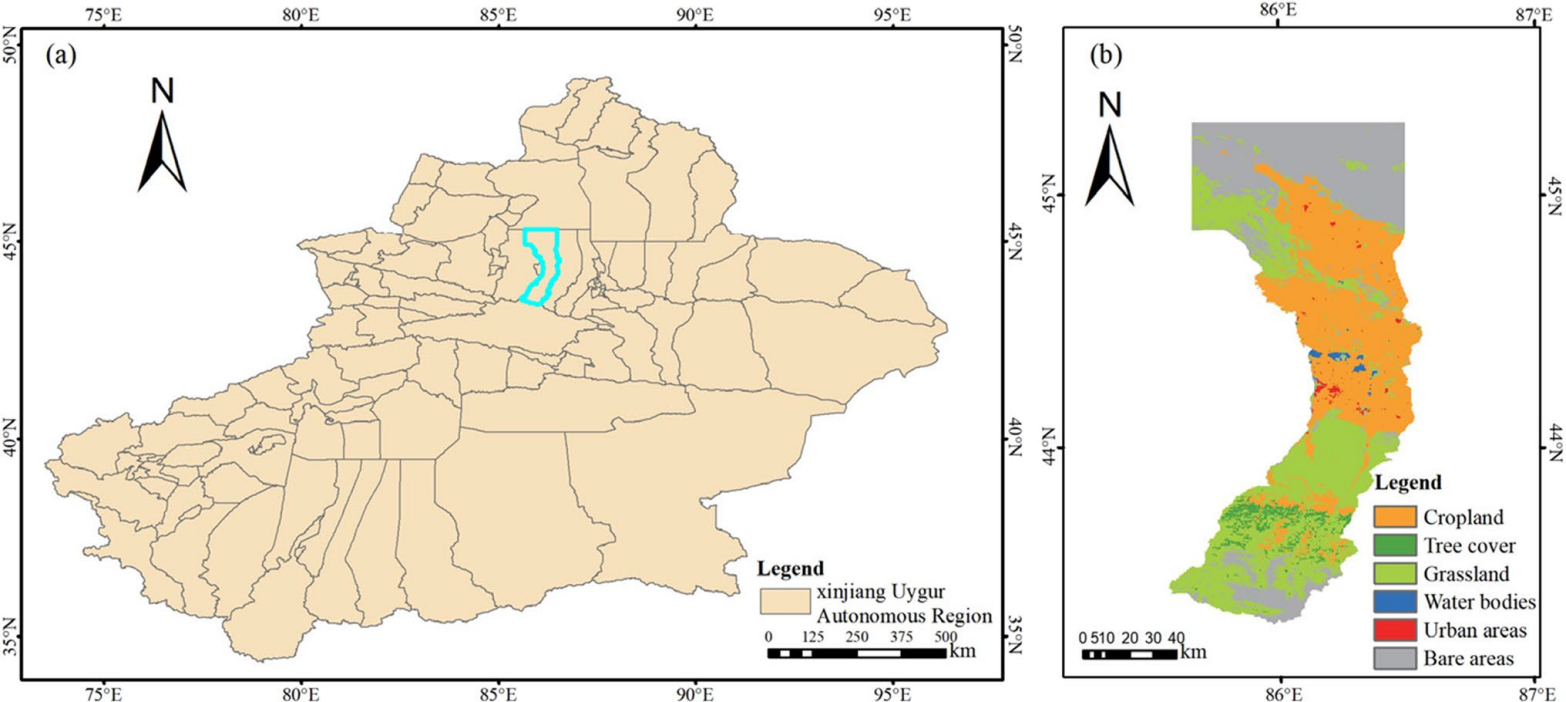
Geographical location and land use pattern of the study area: **a** Xinjiang Uygur Autonomous Region; **b** the Manasi region

The Manasi region, located in the northern part of the Xinjiang Uygur Autonomous Region, has a diverse topography. It lies at 85°40′–86°31′ east longitude and 43°21′–45°20′ north latitude (Fig. 2) and is surrounded by mountains on three sides, situated on the southern edge of the Junggar Basin. The study area has an arid to semi-arid climate, with hot summers and cold winters, and an average annual temperature of around 8.7 °C. There are significant seasonal and diurnal temperature variations, with large differences in temperature between day and night. Precipitation is scarce in the area, with an average annual rainfall of around 237 mm. Most of the precipitation occurs in the summer months, with little to no rainfall during the winter. The vegetation in the study area is mainly desert and steppe vegetation due to the arid climate and rugged topography. The dominant land use categories in the region are cropland, grassland, and bare land, with an occupancy rate of more than 94%. Over the past 30 years, bare land in the region has decreased.

### Data resource and accuracy assessment

The study’s datasets comprised land use data and driving factors (Table 1). The land use data were obtained from the 30-m annual land cover datasets and its dynamics in China from 1990 to 2020 (https://zenodo.org/record/5210928#.Y9TDU3ZBxD). Six land use classes were considered: cropland, tree cover, grassland, water bodies, urban areas, and bare areas.

**Table 1:** Description of the land use data and driving factors used in different land use prediction models

Parameters	Year	Data source	Description
Land use data	1990–2020	The 30-m annual land cover datasets and its dynamics in China from 1990 to 2020 (https://zenodo.org/record/5210928#.Y9TDU3ZBxD)	30 m * 30 m
Population	2010, 2015, 2020	Resource and Environment Science and Data Center (https://www.resdc.cn/)	1 km * 1 km
GDP	2010, 2015, 2020	Resource and Environment Science and Data Center	1 km * 1 km
River map	–	Geographic Information Professional Knowledge Service System (http://kmap.ckcest.cn/resource/search/senior)	–
Road map	2020	Geographic Information Professional Knowledge Service System	–
Soil data	–	Resource and Environment Science and Data Center	1 km * 1 km
DEM	–	Resource and Environment Science and Data Center	12.5 m * 12.5 m
Precipitation	2010, 2015, 2020	National Meteorological Science Data Center (http://data.cma.cn/)	–
Temperature	2010, 2015, 2020	National Meteorological Science Data Center	–
NDVI	2010, 2015, 2020	National Science and Technology Infrastructure (http://www.nesdc.org.cn/sdo/list)	30 m * 30 m

Driving factors in this paper include population, GDP, distances to roads and rivers, soil data, slope, DEM, precipitation, temperature, and NDVI. The slope map was calculated by ArcGIS 10.6 through slope. Distances to roads and rivers were obtained from ArcGIS 10.6 via Euclidean distances. Meteorological data were obtained from the National Meteorological Science Data Center (https://data.cma.cn/), including temperature and precipitation measurements from meteorological stations located in and around the study area for the years 2010, 2015, and 2020. Missing data were interpolated using a linear method. Meteorological raster data were then generated using inverse distance weighting and ensuring that they had the same resolution and projection coordinate system. To ensure consistency across all data, the resolution of each dataset was adjusted to match the land use data’s resolution using the resampling method.

**Fig. 3:**
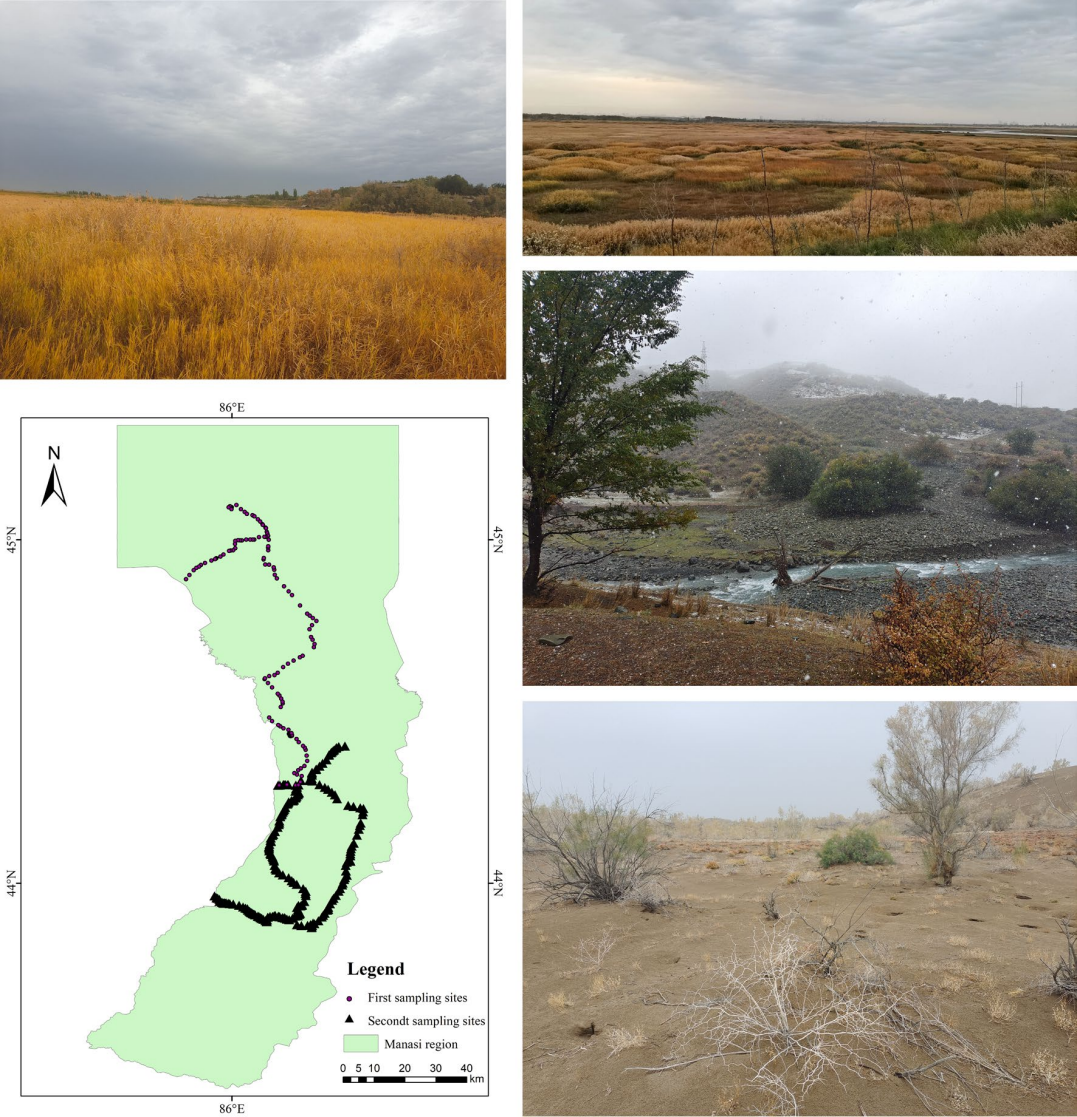
Sample points in the study area and the field survey maps

Field surveys to the study area had been conducted in October 2021. Some ground truth data were collected for accuracy assessment of the land use data using Global Positioning System instruments in the Manasi region (Fig. 3). These data from the field surveys included land use categories and coordinates. The surveys collected 143 and 251 points of experimental data, respectively, of which 358 were valid. In comparison with these points, the results show an accuracy of 92.74% for the land use data in 2020, which were obtained from the 30-m annual land cover datasets and its dynamics in China from 1990 to 2020.

### OLS and Sobol sensitivity analysis

According to established studies and research requirements, ten driving factors were selected, including accessibility factors (distance to rivers, distance to roads), natural factors (DEM, slope, precipitation, soil data, NDVI, temperature), and socioeconomic factors (population density, GDP). However, these factors are multicollinear. OLS is applied to research association between land use data and its drivers (Table 2). The results showed that distance to river, DEM, slope, and NDVI were the main driving factors, with *R*^2^ of 40.79%. The higher the variance inflation factor (VIF), the stronger the covariance; when it is greater than 10, the covariance is considered significant. The VIF value of the four driving factors is all less than 3 which indicates that there is no multicollinearity.

**Table 2:** OLS regression results of the six driving factors to land use in Manasi region

Driving factors	Coefficient	t-Statistic	Probability	VIF
Distance to river	6.220205	1617.436742	0.000000*	1.378559
DEM	0.000147	117.927111	0.000000*	2.878959
Slope	0.0143212	257.603372	0.000000*	2.604565
NDVI	−0.024272	−1457.737635	0.000000*	1.250706

Sensitivity analysis includes both local sensitivity and global sensitivity analysis, which usually represents the change in the output variable when the input variable changes at the nominal value. For non-linear input–output relations, local sensitivity only reflects the sensitivity information of local points, while global sensitivity mainly studies the extent of the uncertainty of the input variable on the output response in its entire distribution domain. The key parameters affecting the output uncertainty can be identified through the global sensitivity analysis, and the Sobol method is one of the most widely used methods in the existing global sensitivity analysis methods, and this paper mainly considers the Sobol method for the global sensitivity analysis.

Due to the large amount of data, a uniform sample was taken using ArcGIS Pro. Then, the Sobol method was used for sensitivity analysis based on the regression function derived from the OLS analysis, which is as follows:

1 $$\begin{array}{c}land\,use\,data\,=\,4.398572\,+\,6.220205\times\,Distance\,to\,river\,+\,0.000147\\\times\,DEM+\,0.0143212\times\,Slope\,-\,0.024272\times\,NDVI\end{array}$$

**Fig. 4:**
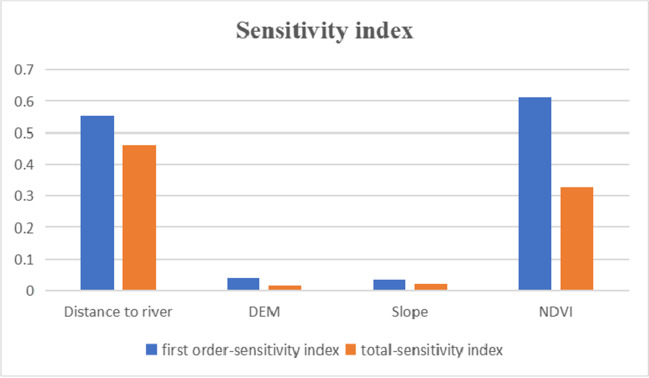
Sensitivity index of land use at 2020 in the Manasi region

Based on the sensitivity analysis results (Fig. 4), the single drivers with the most sensitivity in descending order are NDVI, distance to river, DEM, and slope. For the combined factors, the order of sensitivity magnitude is NDVI, distance to river, slope, and DEM. Both individual and integrated drivers significantly affect land use, with NDVI and distance to rivers having the greatest impact.

### Land use prediction models

#### MLP-LSTM model

**Fig. 5:**
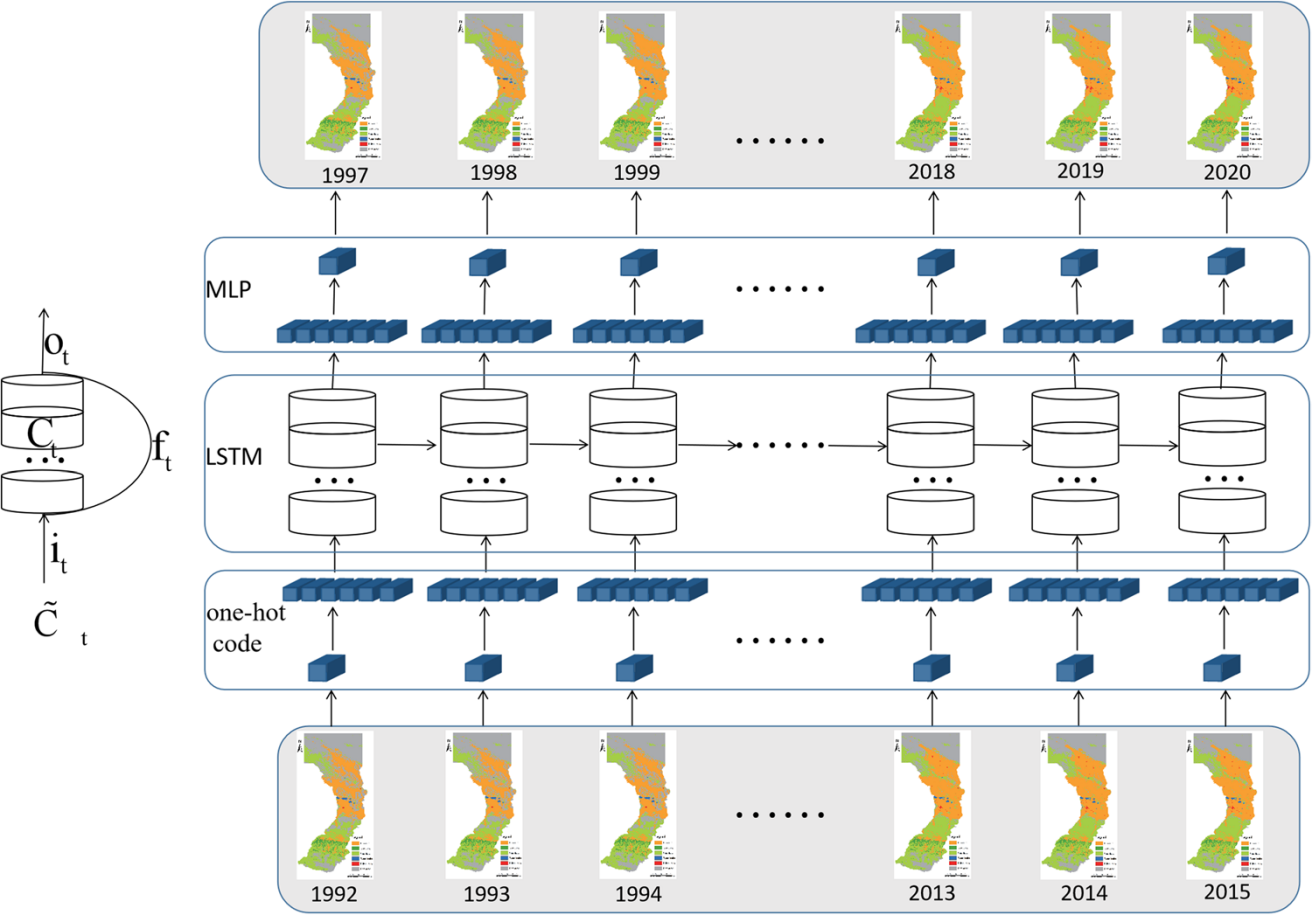
Structure diagram of the MLP-LSTM model

The MLP-LSTM model (Fig. 5) is a type of artificial neural network that includes multiple layers of perceptrons, also known as artificial neurons. During the MLP-LSTM training process, the weights and biases of the neurons are adjusted to minimize the error between the predicted and actual land use changes. The MLP-LSTM model is used to model the transition potential, which indicates the probability of land use change occurring from one land use cover to another. The experiment consisted of 50 iterations using categorical_crossentropy as the loss function, rmsprop as the optimizer, softmax as the activation function, and 64 as the patch size.

The forget gate determines the information that is discarded by the cell unit at the last moment, and the formula is as follows:

2 $${f}_{t}=\sigma \left({W}_{f}g\left[{h}_{t-1},{x}_{t}\right]+{b}_{f}\right)$$

where *ft* is the forget gate, *σ* is the activation function, *Wf* is the weight of the forget gate, *ht − *1 is the state of the unit cell at *t* − 1, *Xt* is the input vector of the cell unit, and *bf* is the bias term of the forget gate.

The input gate determines the stored information of the cell state, which comprises sigmoid and tanh layers. The sigmoid layer determines the vector to be updated, and the tanh layer creates a vector of new candidate values.

3 $$\left\{\begin{array}{l}i_t=\sigma\left(W_ig\left[h_{t-1},x_t\right]+b_i\right)\\{\widetilde C}_t=tanh\left(W_Cg\left[h_{t-1},x_t\right]+b_C\right)\\C_t=f_tgC_{t-1}+i_tg{\widetilde C}_t\end{array}\right.$$

where it is the input gate, ${\widetilde{\mathrm{C}}}_{\mathrm{t}}$ is the new candidate vector, *W*_*i*_ and *W*_*C*_ are weight coefficients, *b*_*i*_ and *b*_*C*_ are bias terms, and *C*_*t*−1_ and *C*_*t*_ are the cell state vectors at *t* − 1 and *t*, respectively.

The output gate controls the influence of long-term memory on the current output. The output gate formula for the unit cells is as follows:

4 $$\left\{\begin{array}{l}o_t=\sigma\left(W_og\left[h_{t-1},x_t\right]+b_o\right)\\h_t=o_t\cdot tanh\left(C_t\right)\end{array}\right.$$

where *o*_*t*_ is the output gate, *W*_*o*_ is the weight of the output gate, *b*_*o*_ is the output gate bias term, and *h*_*t*_ is the unit cell output.

Backward propagation, also referred to as backpropagation, propagates the calculated loss function value forward, calculates the influence of the loss function on each weight or offset, and updates the parameters of the network. Error terms are classified into two types: those that propagate over time and those that propagate along the network layer. The loss function used in this experiment was SoftMax.

The error term that propagates backward over time *k* is calculated as follows:

5 $${\delta }_{k}^{T}=\prod_{j=k}^{t-1}{\delta }_{o,j}^{T}{W}_{oh}+{\delta }_{f,j}^{T}{W}_{fh}+{\delta }_{i,j}^{T}{W}_{ih}+{\delta }_{\widetilde{c},j}^{T}{W}_{ch}$$

where ${\delta }_{o,j}^{T}$, ${\delta }_{f,j}^{T}$, ${\delta }_{i,j}^{T}$, ${\delta }_{\widetilde{c},j}^{T}$ are the error terms of output gate, forget gate, input gate, and current cell input state in *T* period, respectively; ${W}_{oh}$, ${W}_{fh}$, ${W}_{ih}$, ${W}_{ch}$ are weight matrices of output gate, forget gate, input gate, and cell state, respectively.

The error term propagated along the network layer, and the error term transmitted from the *L* − 1 layer to the *L* layer is

6 $$\delta_t^{L-1}=\left(\delta_{o,t}^TW_{ox}+\delta_{f,t}^TW_{fx}+\delta_{i,t}^TW_{ix}+\delta_{\widetilde c,t}^TW_{cx}\right)gf'\left(net_t^{L-1}\right)$$

where ${\delta }_{o,t}^{T}$, ${\delta }_{f,t}^{T}$, ${\delta }_{i,t}^{T}$, and ${\delta }_{\widetilde{c},t}^{T}$ are the error terms of output gate, forget gate, input gate, and current cell input state in *T* period, respectively; ${W}_{ox}$, ${W}_{fx}$, ${W}_{ix}$, and ${W}_{cx}$ are weight matrices of output gate, forget gate, input gate, and cell state, respectively.

The weight gradients include the following formula:

7 $$\begin{array}{l}\frac{\partial E}{\partial {W}_{mh,t}}=\sum_{j=1}^{t}{\delta }_{m,t}{h}_{t-1}^{T}\\ \frac{\partial E}{\partial {W}_{mx}}={\delta }_{m,t}{x}_{t}^{T}\begin{array}{cccc}& & & \end{array}\\ \frac{\partial E}{\partial {b}_{m}}=\sum_{j=1}^{t}{\delta }_{m,j}\end{array}$$

where $\frac{\partial E}{\partial {W}_{mh,t}}$, $\frac{\partial E}{\partial {W}_{mx}}$, and $\frac{\partial E}{\partial {b}_{m}}$ are the weight gradients of ${W}_{mh}$,${W}_{mx}$, and bias, respectively; *m* has four values: output gate, forget gate, input gate, and current cell input state; ${\delta }_{m,t}$ is the error term of *m* at *t* time; ${h}_{t-1}^{T}$ is the output of the unit cell at *T* time; ${x}_{t}^{T}$ is the input vector of the cell unit at *T* time.

#### MLP-ANN model

**Fig. 6:**
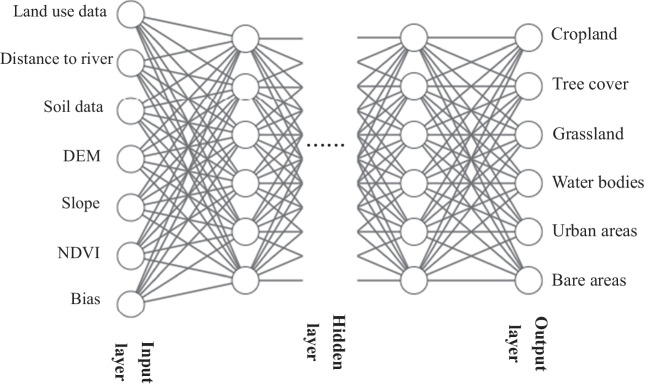
Structure diagram of MLP-ANN model

The MLP-ANN model in the Multi-Objective Land Use Change Evaluation (MOULSCE) module of the QGIS software was used to model the transition potential (Fig. 6), and CA was applied to generate a land use forecast map. The MLP-ANN model is trained using historical data on land use change, as well as a set of input variables that are used to predict future changes in land use (Zeshan et al. 2021). The MLP-ANN training process was iterated 100 times, with a neighborhood value of 8 px and 10 hidden layers. The model’s first step involves using land use maps for the 2010 and 2015 years. Then, the model incorporates driving factors to obtain a land cover change map, establishing the changing pattern for the study area between 2010 and 2015. The plugin calculates the percentage of area change for each year and generates a transition matrix indicating the proportion of area shifting from one land use cover to another. Future land use maps are predicted based on the assumption of existing land use shifting patterns. Additionally, using the land use maps and driving factors of 2015 and 2020, land use transitions are predicted for 2025. The experiment involves 80 maximum iterations, a neighborhood of 64 pixels, a learning rate of 0.16, a momentum of 0.05, and 5 hidden layers.

#### LR model

The LR model is a statistical method used to predict the probability of an event occurring based on a set of explanatory variables. In the MOULSCE module of QGIS, the LR model is used as part of a larger land use change simulation framework. It predicts the probability of a cell transitioning from one land use class to another, and CA is used to simulate land use change. The LR training process was iterated 100 times, with a neighborhood value of 64 px.

8 $$\begin{array}{c}\begin{array}{l}P\left\{\mathrm y\vert\mathrm x\right\}=f(z)=\frac1{1+\mathrm e^{-\mathrm z}}\\z=\beta^{\mathrm T}\chi=\beta_0+\beta_1\chi_1+\beta_2\chi_2+\beta_3\chi_3+...+\beta_{\mathrm n}\chi_{\mathrm n}\end{array}\\\end{array}$$

where *P* represent the probability of the land use category; *y* represents the dependent variable; *x* represents the independent variable; *β*_0_ is the intercept; *β*_1_, *β*_2_, *β*_3_, …, *β*_*n*_ represent the regression coefficient of the independent variable; and *χ*_1_, *χ*_2_, *χ*_3_, …, *χ*_*n*_ are the independent variables representing the driving factors.

#### CA–Markov model

The CA-Markov mode combines the strengths of two well-established modeling techniques: CA and Markov chain modeling. CA is a method used to model spatially explicit systems, such as land use change, by simulating the transition of land use for each cell. The transition rules are typically based on driving factors. Markov Chain is a stochastic process that models the probability of transitioning from one state to another. It is commonly used to model temporal dynamics of a system, such as the transition of land use over time. The CA-Markov model simulates the spatial and temporal dynamics of land use change. The model uses the transition probabilities from the Markov Chain model to simulate the likelihood of a cell transitioning from one land use state to another, based on the current state of the cell and its neighboring cells.

The formula is as follows:

9 $${\mathrm{S}}_{\mathrm{t}+1}={\mathrm{P}}_{\mathrm{ij}}\times {\mathrm{S}}_{\mathrm{t}}$$

where *S*_*t*_ and *S*_*t*+1_ represent the land use category at time *t* and *t* + 1; *P*_*ij*_ represents the probability matrix for land use transfer which is as follows:

10 $${\mathrm{P}}_{\mathrm{ij}}=\left[\begin{array}{cccc}{\mathrm{P}}_{11}& {\mathrm{P}}_{12}& \cdots & {\mathrm{P}}_{1\mathrm{n}}\\ {\mathrm{P}}_{21}& {\mathrm{P}}_{22}& \cdots & {\mathrm{P}}_{2\mathrm{n}}\\ \cdots & \cdots & \cdots & \cdots \\ {\mathrm{P}}_{\mathrm{m}1}& {\mathrm{P}}_{\mathrm{m}2}& \cdots & {\mathrm{P}}_{\mathrm{mn}}\end{array}\right]$$

where *P*_*ij*_ (0 ≤ *P*_*ij*_ ≤ 1) is the probability of conversion of land use category *i* to *j*.

### Model accuracy verification

Confusing the kappa coefficient of the matrix is an effective verification method (Phan et al. 2020; Mohammad et al. 2022). Therefore, the kappa coefficient was applied to verify the accuracy of land use change prediction models. The equation can be shown as follows:

11 $$\mathrm{K}=\frac{{\mathrm{P}}_{\mathrm{o}}-{\mathrm{P}}_{\mathrm{e}}}{1-{\mathrm{P}}_{\mathrm{e}}}$$

where *P*_*o*_ represents the proportion of correct land use predictions; *P*_*e*_ represents the accidental consistency error.

The performance of the land use prediction models is quantitatively measured using commonly used parameters like accuracy, precision, and F1 score. These parameters are shown as follows.

12 $$\mathrm{accuracy}=\frac{TP+TN}{TP+FN+FP+TN}$$

13 $$\mathrm{precision}=\frac{TP}{TP+FP}$$

14 $$\mathrm{F}1=\frac{2*TP}{2*TP+FP+FN}$$

where *TP* and *FP* represent the true and false positives; *TN* and *FN* represent the true and false negatives.

### Landscape indices

Landscape pattern refers to the spatial arrangement and combination of landscape elements with different sizes and shapes. The landscape pattern with regularity can be called the spatiotemporal pattern. Landscape pattern indices are used to explore the temporal and spatial pattern changes of different land use types. In this paper, representative landscape indices are selected, as shown in Table 3, and the differences in landscape indices among different land use prediction models are analyzed and compared.

**Table 3:** Landscape indices and their significance

Landscape Index	Significance
Number of patches (NP)	The larger the NP, the greater the fragmentation of the landscape and the more spatially heterogeneous the features
Landscape shape index (LSI(%))	The larger the LSI, the higher the irregularity of the landscape shape and the higher the spatial heterogeneity
Similarity Proximity Percentage (PLADJ (%))	The larger the PLADJ value, the more aggregated and adjacent the patch type is in the landscape. The range of values for PLADJ is from 0 to 100
Dispersion and Concurrency Index (IJI (%))	Higher IJI values indicate the proximity of the same plaques
Separation index (SPLIT (%))	Reflecting the degree of landscape fragmentation, this index indicates the extent to which a patch is disturbed. The higher the index value, the greater the degree of fragmentation
Landscape Segmentation DIVISION (%)	Reflecting the fragmentation of the landscape and the complexity of its spatial structure
Aggregation Index (AI (%))	The higher the index, the more compact the patch structure of the same type in the landscape
Modified Simpson's evenness index (MSIEI)	Reflecting the evenness of the landscape, the more even the landscape, the higher the index
Modified Simpson’s diversity index (MSIDI)	Reflecting landscape diversity, the richer the landscape, the higher the index
Shannon’s diversity index (SHDI)	Reflecting landscape heterogeneity, the SHDI is more sensitive to the uneven distribution of patch types in the landscape, emphasizing the contribution of rare patch types to the information. A higher SHDI value indicates greater landscape heterogeneity, which is typically associated with a richer land use and higher fragmentation
Maximum Plaque Index (LPI (%))	The dominance of landscape types in the composition of the landscape can be determined by the LPI value, with higher values indicating a greater degree of human activity disturbance

## Results

### Analysis of land use change in Manasi from 1990 to 2020

**Fig. 7:**
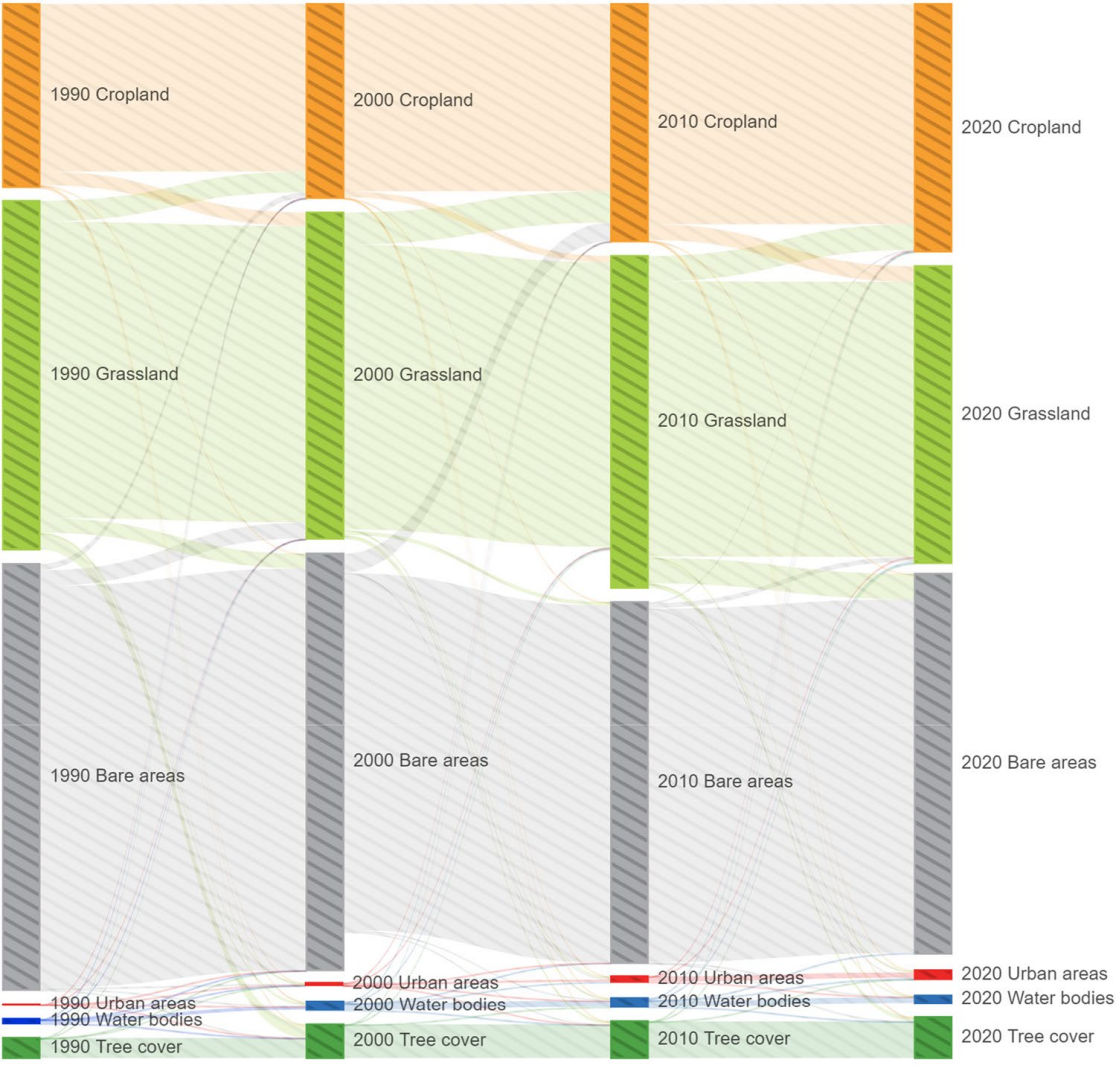
Sankey diagram of land use area from 1990 to 2020 in the Manasi region

**Fig. 8:**
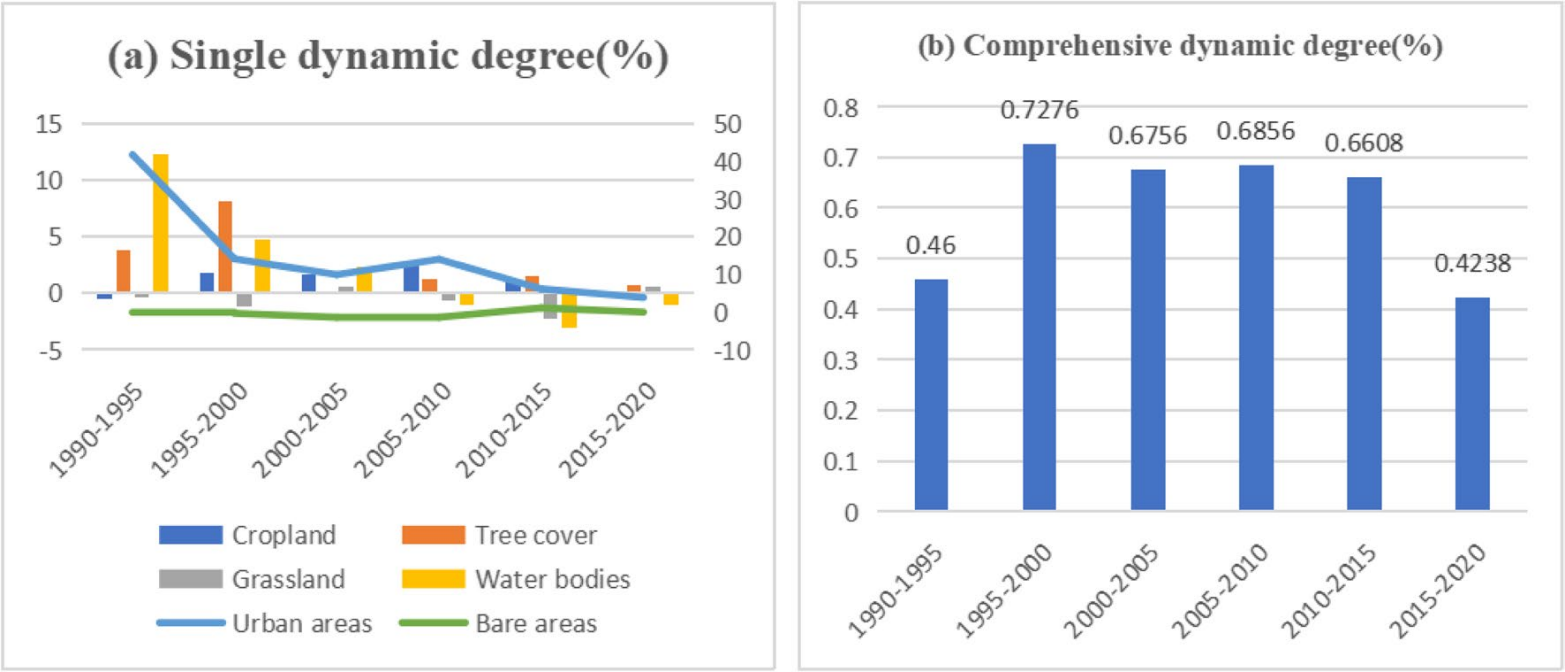
Dynamic degree of land use at different time points in the Manasi region: **a** single dynamic degree; **b** comprehensive dynamic degree

According to the Sankey diagram (Fig. 7) and the dynamic degree of land use (Fig. 8), in 1990, 2000, 2010, and 2020, the proportions of cropland were 18.62%, 19.81%, 24.22%, and 25.27%, respectively. According to the Sankey diagram (Fig. 6), the growth in cropland from 1990 to 2010 was mostly because of conversion of grassland into cropland. The single dynamic degree showed a slowdown in the growth of cropland from 2015 to 2020. The cropland growth trend from 2005 to 2010 was faster than that of the previous 5 years, which is attributed to the implementation of the Tianshan North Slope Economic Zone land preparation project from 2006 to 2010. Subsequently, the cropland area decreased and was replaced by grassland, urban areas, and tree cover, which is closely related to the Green Grain Project (GFGP) and urban expansion.

The growth rate of tree cover in the study area has been slow from 1990 to 2020, and it has not increased significantly since the implementation of the GFGP. This can be attributed to the climate and landform characteristics of the study area. The study area can be divided into three parts based on its landform: mountains, plains, and deserts. Since mountains and deserts are not suitable for tree cover growth. Moreover, the water resources in the plains mainly come from the Manasi River and alpine glacier melt water. Trees struggle to survive in this fragile ecology.

The grassland in Manasi has been an important land use type that can adapt to the dry environment. However, the grassland showed a decrease in trend from 1990 to 2020. In 1990, 2000, 2010, and 2020, the proportions of grassland were 35.44%, 33.20%, 33.73%, and 30.18%, respectively. The grassland mainly converted to cropland, tree cover, and bare areas.

The water bodies increased by 122.52% from 1990 to 2005 and followed by a slow decrease until 2020 with a decrease of 23.83%. The area of bare land has been in decline except for 2010 to 2015. It is noteworthy that the conversion of cropland, grassland, and bare land to urban areas has been significant, indicating rapid urbanization in the region. Cropland, grassland, and bare land are converted to urban areas, accounting for 33.37%, 31.25%, and 28.13% of the total conversion area, respectively. Urban areas increased by 1997.15% from 1990 to 2020. These growth rates show that urban areas are in an expansion period.

### Prediction of land use in Manasi region

The 2020 maps were predicted based on 2015 probabilities by using MLP-LSTM, MLP-ANN, LR, and CA-Markov models and then compared that 2020 predicted map with their original results and calculated the kappa coefficients which were calculated to be 95.58%, 93.36%, 89.48%, and 85.35%, respectively. According to the kappa coefficient, the LSTM model has a good simulation effect and high reliability. The accuracy, precision, and F1 scores of all methods are shown in Table 4. In general, it can be found that the MLP-ANN and MLP-ANN models obtain higher accuracy in most levels, while the CA–Markov model has the lowest accuracy.

**Table 4:** Accuracy metrics for the land use prediction models (%)

Land use types	MLP-LSTM model			MLP-ANN model			LR model			CA–Markov model		
	Accuracy	Precision	F1	Accuracy	Precision	F1	Accuracy	Precision	F1	Accuracy	Precision	F1
Cropland	98.75	97.43	96.51	97.45	94.32	95.10	91.58	79.09	85.14	91.35	83.37	84.36
Tree cover	99.78	99.89	97.45	99.84	99.92	98.08	99.78	99.93	97.64	98.88	92.60	87.84
Grassland	98.64	94.25	90.93	95.94	93.94	93.25	89.15	89.25	80.40	86.84	76.88	79.53
Water bodies	99.94	84.63	85.36	99.71	80.36	82.27	99.47	73.32	65.43	99.28	56.51	67.81
Urban areas	99.43	75.46	73.01	99.42	64.74	72.53	99.70	94.00	83.31	99.09	55.44	59.86
Bare areas	98.64	98.13	99.27	98.26	97.95	97.81	96.37	93.70	95.80	91.30	92.80	89.22

**Fig. 9:**
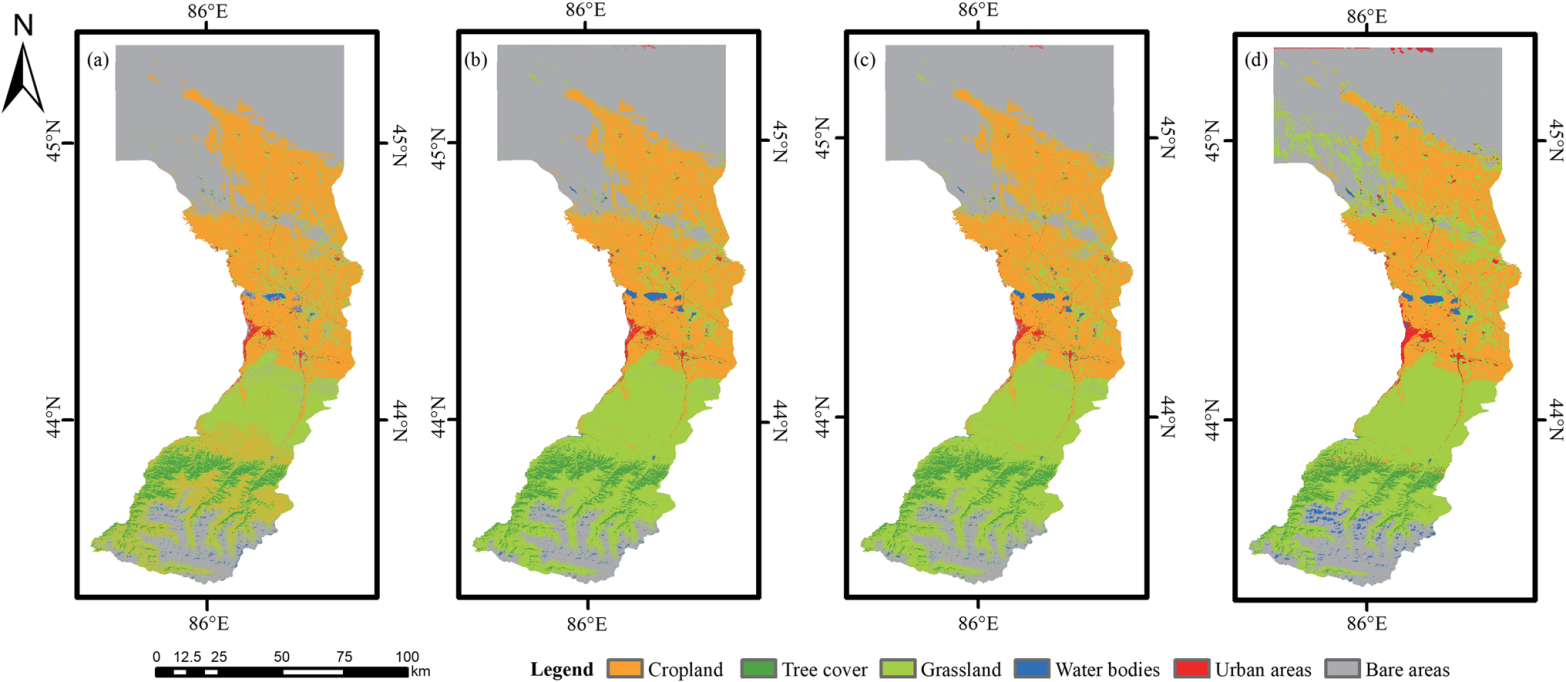
Spatial distribution of prediction land use of models in 2025. (a) MLP-LSTM model, (b) MLP-ANN model, (c) LR model, (d) CA–Markov model

According to the Sankey diagram (Fig. 7) and the prediction results for land use in 2025 (Fig. 9), land use change tends to be stable. By 2025, the land use prediction results reveal that the land use types are still mainly cropland, grassland, and bare land, with cropland and urban areas increasing; tree cover, grassland, and bare land decreasing; and the area of water bodies remaining largely unchanged.

In the Manasi region, bare land, such as desert and mountainous areas, accounted for 29.06% of the total area, whereas tree cover accounted for only 2.57%. Tree cover and grasslands are important ecological protection barriers that play an important role in reducing soil erosion, preventing wind, and fixing sand. In addition, the Manasi region is arid and rainless, and its ecology is fragile, indicating that environmental protection and ecological barriers are facing severe challenges. The implementation of the GFGP policy had positive effects on the area of tree cover and grass. Based on practical protection of cropland, cropland should be returned to tree cover and grassland, which not only ensures food security but also helps improve the ecological quality of the environment.

### Comparative analysis of prediction methods based on landscape indices

Landscape indices were employed to evaluate the spatial configuration of land use projections. Seventeen indices in landscape level and six indices in class level were calculated by Fragstats 4.2 software. The landscape indices in the landscape level for each model predicting land use are shown in Table 5. The CA–Markov model shows the lowest overall number of patches and the smallest LSI, indicating that the model predicts low land use landscape shape and complexity. The LR model exhibited the highest overall number of patches.

**Table 5:** Landscape indices for different land use prediction models in 2025 in the landscape level

Models	NP	LSI	PLADJ	IJI	SPLIT	DIVISION	AI	MSIEI	MSIDI	SHDI
MLP-LSTM model	43317	57.1355	94.8565	60.5648	26.3154	0.9616	94.9875	0.6574	1.1849	1.2768
MLP-ANN model	45745	57.8429	94.8025	60.5061	26.2707	0.9619	94.8597	0.6604	1.1832	1.2895
LR model	73716	72.0741	93.5156	57.3624	30.2468	0.9669	93.5720	0.6595	1.1816	1.2875
CA–Markov model	15973	39.6699	97.0846	64.5928	21.0660	0.9525	97.1389	0.6727	1.2053	1.3210

**Fig. 10:**
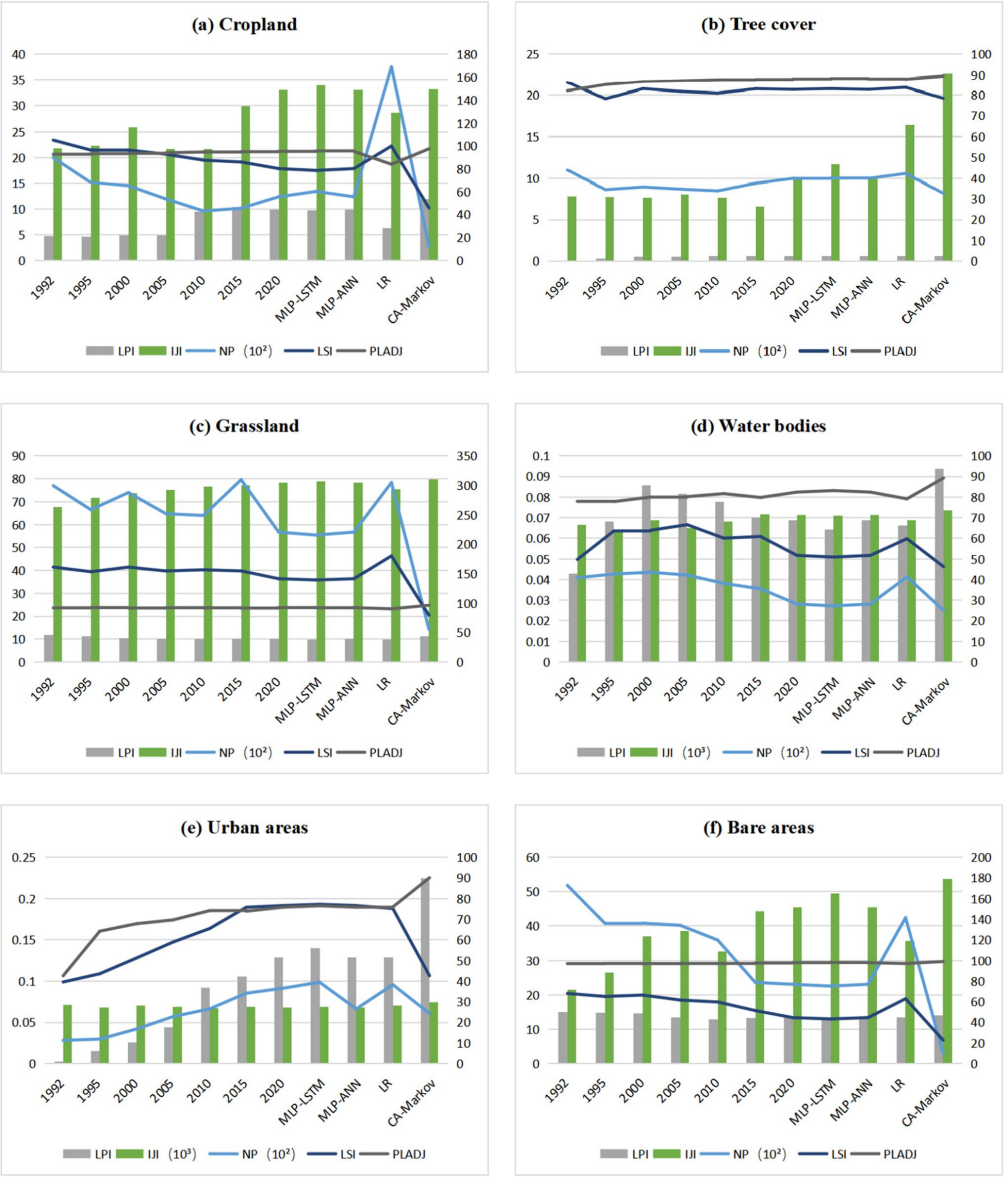
Changes in the landscape pattern indicators of six land use categories in the Manasi region (The changes in the LPI, TE, and NP refer to the primary coordinate axis, and the changes in the LSI, PLADJ, and IJI refer to the secondary coordinate axis)

The landscape indices in class level for each land use type were predicted by four models (Fig. 10). In the CA–Markov model, the NP and LSI for the six land use categories were the smallest, indicating the lowest degree of patch fragmentation and patch shape complexity. Among the four land-use prediction models, the CA–Markov model showed the largest PLADJ indices, indicating that the model predicted the highest patch aggregation. The patch-level landscape index, CA-Markov, is different from other models, with higher aggregation.

From the overall trend of landscape indices, it can be observed that the indices of the CA-Markov and LR models, except for urban areas, are more variable than those of the other models from 1990 to 2020 and less consistent with the overall trend of the landscape indices. The overall indices of the MLP-LSTM and MLP-ANN models were similar and consistent with the trend from 1990 to 2020.

## Discussion

The disorderly development of land resources has caused ecological problems, such as ecological fragility and reduction of biodiversity, which severely restrict the long-term development of land use. Based on a large number of remote sensing data, this paper takes Manasi as the research object to compare and analyze the prediction effect of four land use prediction models.

The kappa coefficient, accuracy, precision, and F1 were utilized to verify the accuracy and reliability of the land use prediction results. The kappa coefficients of the land use prediction results obtained in this study were above 0.90. Particularly, the coefficients of the MLP-LSTM and MLP-ANN models achieved 95.58% and 93.36%, respectively. Compared with related studies (Mokarram and Pham n.d; Song et al. 2020; Zhang et al. 2022a, b; Zhang et al. 2022a, b; Wang et al. 2022), the accuracy of the land use prediction results of the MLP-LSTM model is clearly better. In previous studies (Abdullah and Nakagoshi 2006; Shen et al. 2020; Li et al. 2021), many scholars used landscape indices to describe land use spatial change. Landscape indices are highly concentrated landscape pattern information, which reflects the structural composition and spatial allocation characteristics of each land use type (Yang et al. 2022). Therefore, landscape indices are taken as an index to compare the prediction results.

Even when different models assessed the same scenario, model projections varied substantially (Sohl et al. 2016). The landscape indices pertaining to different land use types at the class level, as projected by four models, reveal that the CA-Markov and LR models exhibit greater variability in indices compared to the other models, with the exception of urban areas, between 1990 and 2020. Moreover, these indices are not in line with the overall trend of landscape indices. CA-Markov needs more parameters in the land use simulation. Most studies are used for multi-scenario land use simulation, and less studies are used for land use prediction. Therefore, the model performs better in the multi-scenario simulation, which could help policymakers in planning future LULC strategies that are sustainable and environmentally friendly. Conversely, the overall indices of the MLP-LSTM and MLP-ANN models show similarity and consistency with the trend from 1990 to 2020.

However, the four models used in this study show low prediction results for urban areas. The F1 scores for the CA-Markov and LR models were particularly low, while the MLP-ANN and MLP-LSTM models, which had higher overall accuracy, still only achieved F1 scores of around 73%. This seems to be because urban areas are more affected by human activities and policies, as confirmed by other studies (Xu et al. 2022; Zhang and Bin 2022). To improve the accuracy of land use predictions, future researches need these factors. Additionally, while most current land use prediction studies focus on a single model, it would be beneficial to compare the performance of multiple models.

## Conclusions

Using the Manasi region as the research object and combining GIS and RS, in this paper, the characteristics of land use changes from 1990 to 2020 were analyzed. The precision of the MLP-LSTM, MLP-ANN, LR, and CA-Markov models was compared, and land use changes were predicted in 2025. The findings are as follows:

1. The land use changes were analyzed from 1990 to 2020. The land use changes are as follows: cropland > grassland > bare land > tree cover > urban areas > water areas. The largest change rate of land use area is in urban areas, at 1997.15%. This was followed by tree cover and bare land, with 103.65% and 69.49%, respectively. Tree cover increased, whereas grassland and water bodies decreased. The growth in cropland area was mostly because of the large-scale transfer of bare land. Although urban areas in the study area occupy a small area, it constantly encroaches on the surrounding bare land, cropland, and grassland areas.
2. The MLP-LSTM, MLP-ANN, LR, and CA-Markov models were compared and analyzed. These models predicted land use in 2020 and verified its accuracy. The spatial pattern of land use was predicted in 2025. The MLP-LSTM model had the highest Kappa coefficient at 95.58%, indicating good prediction performance compared to other models. Based on the MLP-LSTM prediction results for 2025, cropland, grassland, and bare areas are expected to remain the main land use types, with an increase in cropland and urban areas. Tree cover, grassland, and bare areas are expected to decrease, while water bodies are expected to remain unchanged.
3. According to the overall trend of landscape indices, except for urban areas, the landscape indices of the land use prediction results of CA-Markov and LR models have changed significantly compared with those of other models from 1990 to 2020, which is not in line with the landscape index development trend. The landscape indices of the land use prediction results of MLP-LSTM and MLP-ANN models were similar, which is in line with the changing trend from 1990 to 2020.

## Data Availability

The datasets generated during and/or analyzed during the current study are available from the corresponding author on reasonable request.
